# Inflammasomes and Type 1 Diabetes

**DOI:** 10.3389/fimmu.2021.686956

**Published:** 2021-06-09

**Authors:** James Alexander Pearson, F. Susan Wong, Li Wen

**Affiliations:** ^1^ Diabetes Research Group, Division of Infection and Immunity, School of Medicine, Cardiff University, Cardiff, United Kingdom; ^2^ Section of Endocrinology, Internal Medicine, School of Medicine, Yale University, New Haven, CT, United States

**Keywords:** inflammasomes, microbiota, type 1 diabetes, NOD mice, humans

## Abstract

Microbiota have been identified as an important modulator of susceptibility in the development of Type 1 diabetes in both animal models and humans. Collectively these studies highlight the association of the microbiota composition with genetic risk, islet autoantibody development and modulation of the immune responses. However, the signaling pathways involved in mediating these changes are less well investigated, particularly in humans. Importantly, understanding the activation of signaling pathways in response to microbial stimulation is vital to enable further development of immunotherapeutics, which may enable enhanced tolerance to the microbiota or prevent the initiation of the autoimmune process. One such signaling pathway that has been poorly studied in the context of Type 1 diabetes is the role of the inflammasomes, which are multiprotein complexes that can initiate immune responses following detection of their microbial ligands. In this review, we discuss the roles of the inflammasomes in modulating Type 1 diabetes susceptibility, from genetic associations to the priming and activation of the inflammasomes. In addition, we also summarize the available inhibitors for therapeutically targeting the inflammasomes, which may be of future use in Type 1 diabetes.

## Introduction

**Figure 1:**
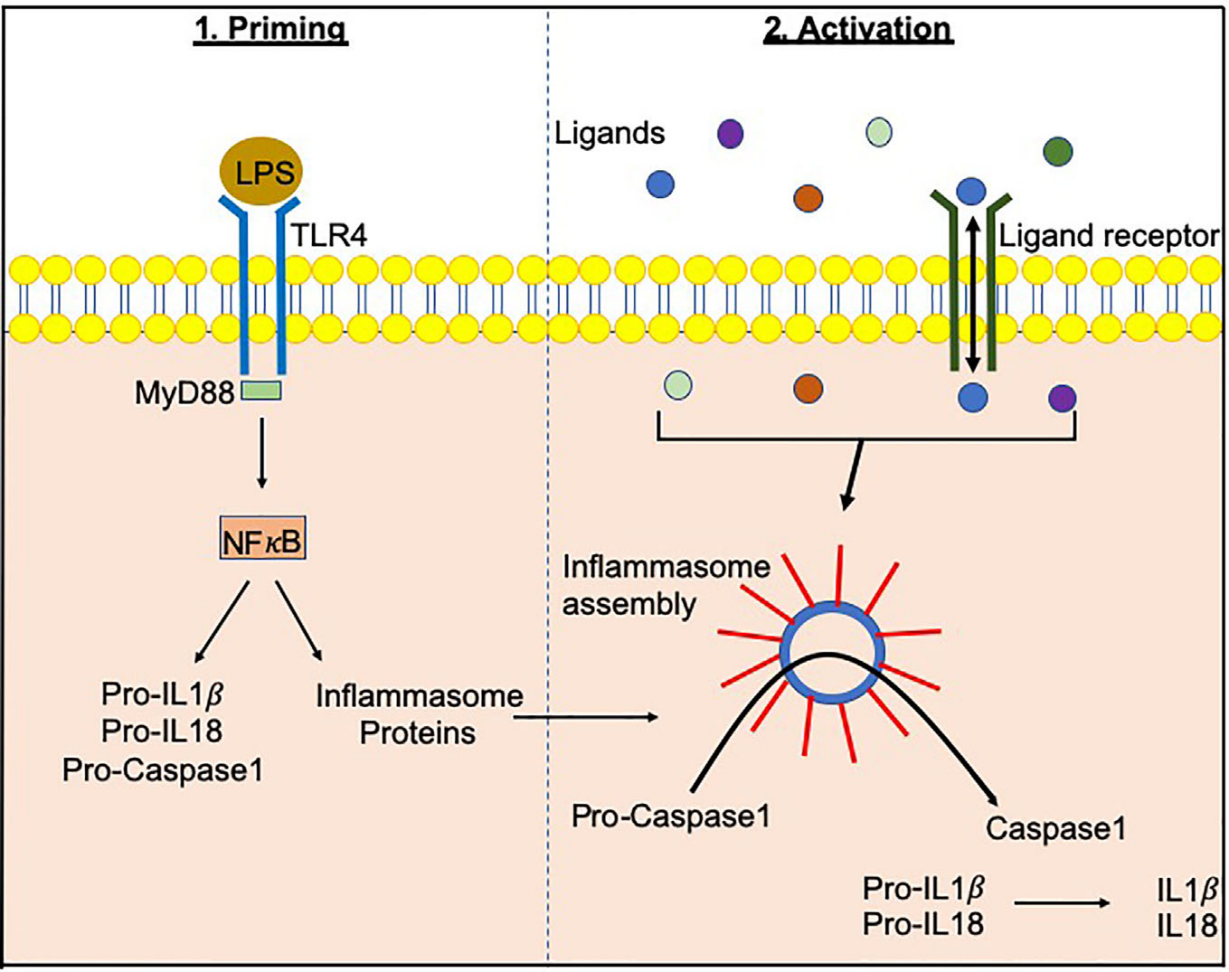
Inflammasome priming and activationInflammasome-related genes e.g. NLRP3, NLRC4 are transcribed following PAMP/DAMP recognition by their respective receptors e.g. bacterial Lipopolysachharide (LPS) recognition by TLR4 pathogen-associated molecular patterns. This “priming” step alerts the cells to potential dangers and prepares the inflammasome machinery to be translated. Upon recognition of additional activating signals (**Figure 2**), the inflammasome proteins oligomerize and form a wheel/disk-like structure. The formation of these inflammasome complexes enables the activation of caspase 1 from its precursor form (procaspase 1), which in turn activates other cytokines including IL-1β and IL-18 (5, 6). Inflammasome-associated proteins can also activate other caspases including caspase 4, 5, 8 and 11 (7–20).

**Figure 2:**
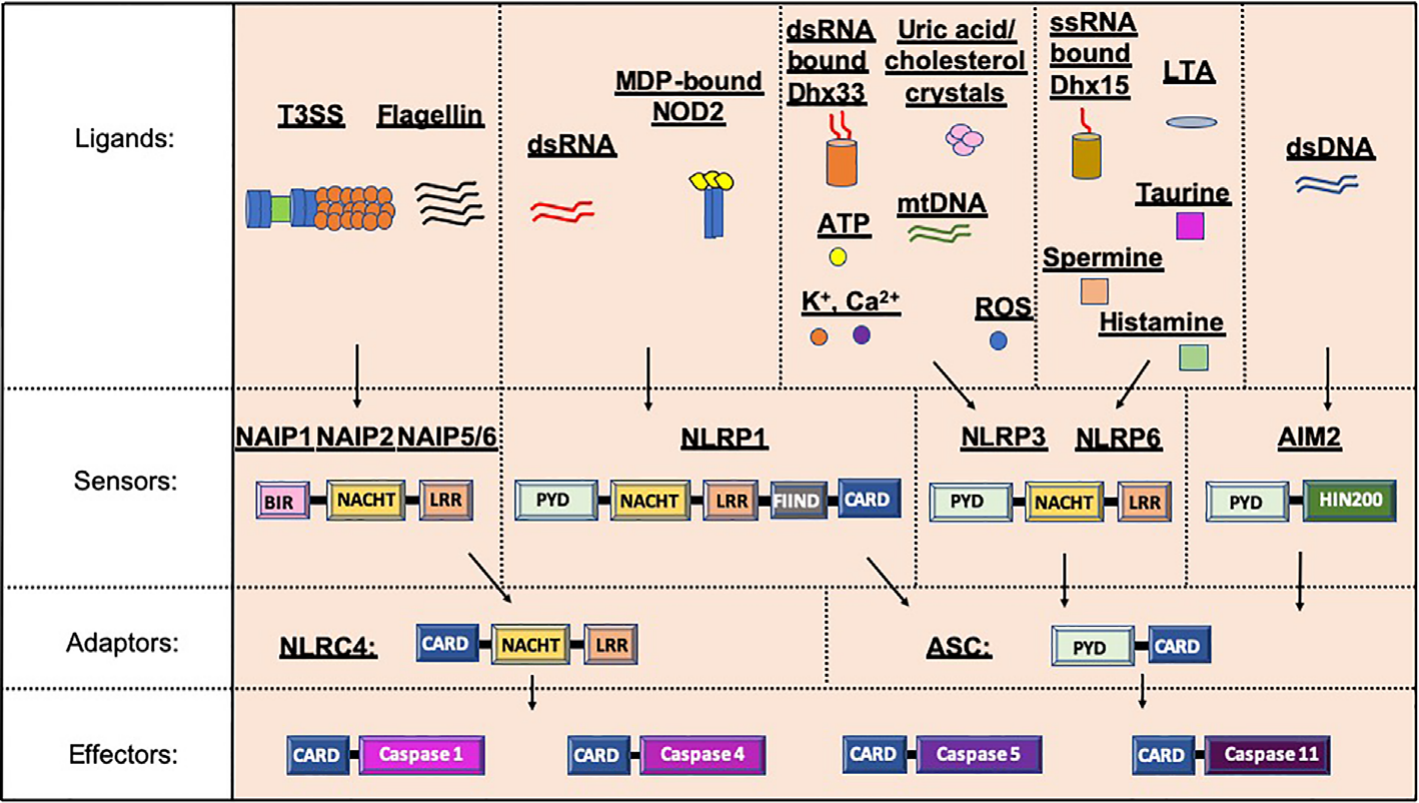
Inflammasome protein sensors and adaptors recognize a variety of ligands, either directly or indirectly. Upon ligand binding, the sensors and adaptors interact via PYD-PYD domain interactions to form the oligomers prior to ASC-mediated recruitment of the Procaspase via CARD-CARD interactions (5–11, 21, 25, 31, 32). NAIP1, 2 and 5/6 bind bacterial-derived Type 3 Secretion system (T3SS) rod or needle proteins or flagellin respectively, prior to activation of the NLRC4 inflammasome (12, 13). NLRP1 can be activated by double stranded RNA (dsRNA; human only) or muramyl dipeptide (MDP) bound to the Nuclear oligomerization domain-containing 2 (NOD2) protein (14, 33). Numerous ligands for NLRP3 have been found including K+, Ca2+, reactive oxygen species (ROS), Adenosine triphosphate (ATP), uric acid crystals, cholesterol crystals, double-stranded RNA (dsRNA) bound by DExD/H-box helicase (Dhx) 33 and mitochondrial DNA (mtDNA) (7, 16, 34–37). Single stranded RNA (ssRNA) bound to Dhx15, lipoteichoic acid (LTA) as well as spermine, taurine and histamine can all activate the NLRP6 inflammasome (32, 35, 38). To date, double stranded DNA is the only ligand known for AIM2 (10, 19, 20). PYD, Pyrin domain; HIN200, Hematopoietic expression, interferon-inducible nature, and nuclear localization 200 domain; NACHT, Nucleotide binding and oligomerization domain; LRR, Leucine-rich repeat; FIIND, function to find domain; CARD, Caspase recruitment domain; BIR, Baculovirus IAP-repeat domains.

Inflammasomes, a term first coined by Dr. Jurg Tschopp in 2002, are multiprotein complexes found in the cytosol, which mediate the activation of inflammatory caspases (1). Inflammasome formation is driven (“primed”) by activation of the pattern-recognition receptors (PRRs) in response to pathogen-associated molecular patterns (PAMPs) or damage signals (e.g. damage-associated molecular patterns that are also known as danger-associated molecular patterns, DAMPs) in the cytosol (2–4) (**Figure 1**). In some inflammasomes, the inflammasome adaptor protein designated as Apoptosis-associated Speck-like protein, containing a Caspase activation and recruitment domain (ASC), aids in the oligomerization of the inflammasome components and links the upstream inflammasome sensor molecules to procaspase 1 (21). In ASC-independent inflammasomes, interactions occur between inflammasome components, which can alter the protein structure e.g. NLRC4 can be activated by Neuronal apoptosis inhibitory proteins (NAIPs), resulting in the formation of the disk-like inflammasome (22, 23). In both ASC-dependent and -independent inflammasomes, procaspase 1 becomes dimerized and through autoproteolysis forms catalytically-active caspase 1, which subsequently induces IL-1β and IL-18 cytokine release, as well as inducing pyroptosis, a form of lytic cell death. There are many different types of proteins involved in the formation of the inflammasomes, including the NBD leucine-rich repeat-containing receptor (NLR) family (e.g. NLRP1) and the PYHIN protein families [e.g. absent in melanoma 2 (AIM2)]. In humans, there are 22 NLRs but only NLRP1, NLRP3, NLRP6, NLRP7, NLRP12 and NLRC4 have been shown to form inflammasomes (24–30). Structural and functional differences between the inflammasome proteins result in differences in their ability to bind their respective ligands, and thus each can be activated by different mechanisms (**Figure 2**). In the case of NLRP3, multiple types of ligands can be recognized, which induce disassembly of the trans-Golgi network, leading to the recruitment and binding of NLRP3 *via* its lysine motif (between the PYRIN and NACHT domain) to the phosphatidylinositol-4-phosphate on the disassembled *trans* face of the golgi (39). However, it is unclear whether there are additional mechanisms, including the question of whether other factors contribute to the Golgi network disassembly, or protection from disassembly, or whether similar mechanisms exist for other inflammasomes.

Inflammasomes can be activated by a number of components released during cell/tissue damage, metabolism, infection or by commensal bacteria. Microbial ligands from host commensals or infectious organisms e.g. type 3 secretion system proteins, flagellin, and DNA/RNA can all activate inflammasome proteins. Furthermore, aggregates of Lipopolysaccharides (LPS; specifically, the Lipid A component), an endotoxin present in the outer membrane of gram-negative bacteria, can directly bind to and activate non-canonical inflammasome caspases 4 and 5 (humans) and 11 (mice) (40–43). Importantly, this process 1) is independent of Toll-like receptor (TLR) 4, which can also bind LPS (40, 42), and 2) promotes protection from cytosolic invading pathogens (40–43). Together, these suggest an important role for microbial modulation of inflammasome responses.

Studies using inflammasome-deficient mice have demonstrated that inflammasomes can influence disease susceptibility to inflammatory bowel disease (IBD) (27, 44), cancer (44, 45), obesity (46, 47), viral/bacterial infection (38, 48–53) and type 1 diabetes (T1D) (34, 54, 55). To date, few studies have functionally investigated the mechanistic role of inflammasomes in T1D; however, there are studies indicating a link to inflammasomes and susceptibility to T1D. As susceptibility to T1D can be modulated by microbial components, as discussed later, we highlight the role of inflammasomes as important microbial sensors in the context of T1D.

## Single Nucleotide Polymorphisms Link Inflammasomes to Type 1 Diabetes Susceptibility

Genetic analyses often provide important insight into genes or mutations that may be associated with disease susceptibility in humans. Gene mutations in NLRP3, resulting in a gain of function and thus increased IL-1β secretion, were initially linked to a number of inherited autosomal dominant inflammatory diseases e.g. Muckle-Wells syndrome and familial cold autoinflammatory syndrome and chronic infantile neurological cutaneous articular syndrome (56). Since then, single nucleotide polymorphisms (SNPs) in *NLRP1*, *NLRP3* and *NLRC4* have been associated with many autoimmune diseases including IBD (57), celiac disease (58), multiple sclerosis (59) and autoimmune diabetes (60–64). **Table 1** summarizes the SNPs in *NLRP1*, *NLRP3* and *NLRC4* genes that have been investigated in individuals with Type 1 diabetes. Of these SNPs, only 2 are within the coding region of *NLRP1* and *NLRP3* genes (rs12150220 and rs35829419 respectively) and both have been linked to a gain of function and excessive IL-1β and IL-18 secretion in other disease settings (67, 68). The other SNPs that are located in the promoter region may influence gene regulation, but this has not yet been fully elucidated. As **Table 1** illustrates, not all populations studied show the same SNP associations in individuals with Type 1 diabetes. For example, the SNP rs12150220, located in the *NLRP1* gene region, was increased in a Norwegian population with T1D (60); however, no associations were identified in either a Polish (65) or Brazilian (62) population with T1D, compared to their controls. There may be many reasons for this, including population-based genetic differences, the presence of other comorbidities or the microbiota composition. Two studies conducted in the Han Chinese population also showed SNP associations in *NLRP3* and *NLRC4* gene regions with clinical characteristics, including the age of diabetes onset, 2-hour postprandial c-peptide and the presence of anti-glutamic acid decarboxylase (GAD) autoantibodies (63, 66). These suggest a potential link to altered immunity; however, larger scale studies are needed to help us to better understand the association of different allelic variants and combinations of haplotypes in the inflammasome-related genes and susceptibility to Type 1 diabetes. Studies using knock-in mice, in which the SNPs can be introduced into the gene, may provide valuable tools to elucidate the functional consequences of these SNPs.

**Table 1:** SNPs in inflammasome genes that have been investigated for associations with autoimmune diabetes in humans.

Gene and location	SNP (and alleles)	Study population	Association	Reference
*NLRP1* (17p13.2)	rs12150220 (T/A)	Norwegian population; T1D: n=1086 with disease onset before 17 years of age; Controls n=3273	rs12150220 increased in individuals with T1D vs controls - OR=1.16, p=0.006	(60)
	rs6502867 (C/T)			
	rs2670660 (G/A)		No differences between individuals with T1D and controls in any of the other SNPs	
	rs878329 (C/G)			
	rs6502867 (G/A)	Polish population; T1D: n=221 with disease onset before 13 years of age; Controls: n=254	No differences between individuals with T1D and controls in any of the SNPs	(65)
	rs12150220 (T/A)			
	rs2670660 (T/C)			
	rs878329 (C/G)			
	rs8182352 (A/G)			
	rs4790797 (C/T)			
	rs12150220 (A/T)	Pediatric Brazilian population; T1D: n=196 (n=136 with T1D only, n=50 with T1D and Celiac disease and/or Thyroiditis); Controls n=192	No differences between individuals with T1D and controls in any of the SNPs	(62)
	rs2670660 (G/A)			
	rs11651270 (C/T)	Chinese Han population; T1D: n=510; Sex-matched controls n=531	rs11651270 CT frequency lower in T1D population vs controls – OR=0.714 p=0.002	(63)
	rs2670660 (G/A)			
			rs2670660 GA frequency lower in T1D population vs controls – OR=0.706 p=0.026	
			rs11651270 TT genotype associated with younger age at onset vs rs11651270 CT and CC genotypes in T1D cohort p=0.001	
*NLRP3* (1q44)	rs10754558 (C/G)	Pediatric Brazilian population; T1D: n=196 (n=136 with T1D only, n=50 with T1D and Celiac disease and/or Thyroiditis); Controls n=192	rs10754558 G minor allele frequency lower in T1D population vs controls p=0.004	(62)
	rs35829419 (C/A)			
	rs10802501 (T/A)			
			No differences between individuals with T1D and controls in the other SNPs.	
*NLRC4* (2p22.3)	rs212704 (T/C)	Chinese Han population; T1D: n=510; Sex-matched controls n=531	No differences between individuals with T1D and controls in any of the SNPs	(66)
	rs385076 (C/T)			
			rs212704 genotype vs 2 hour postprandial c-peptide, p=0.003	
			rs385076 genotype vs Onset age, p=0.031	
			rs385076 genotype vs GADA+ (%), p=0.041	

rs12150220 and rs35829419 SNPs encode coding sequence variants. Many of the other SNPs are located within the promoter regions.OR, Odds Ratio at 95% confidence interval.

## Altered Microbial Composition May Drive Inflammasome Activation in Type 1 Diabetes

**Figure 3:**
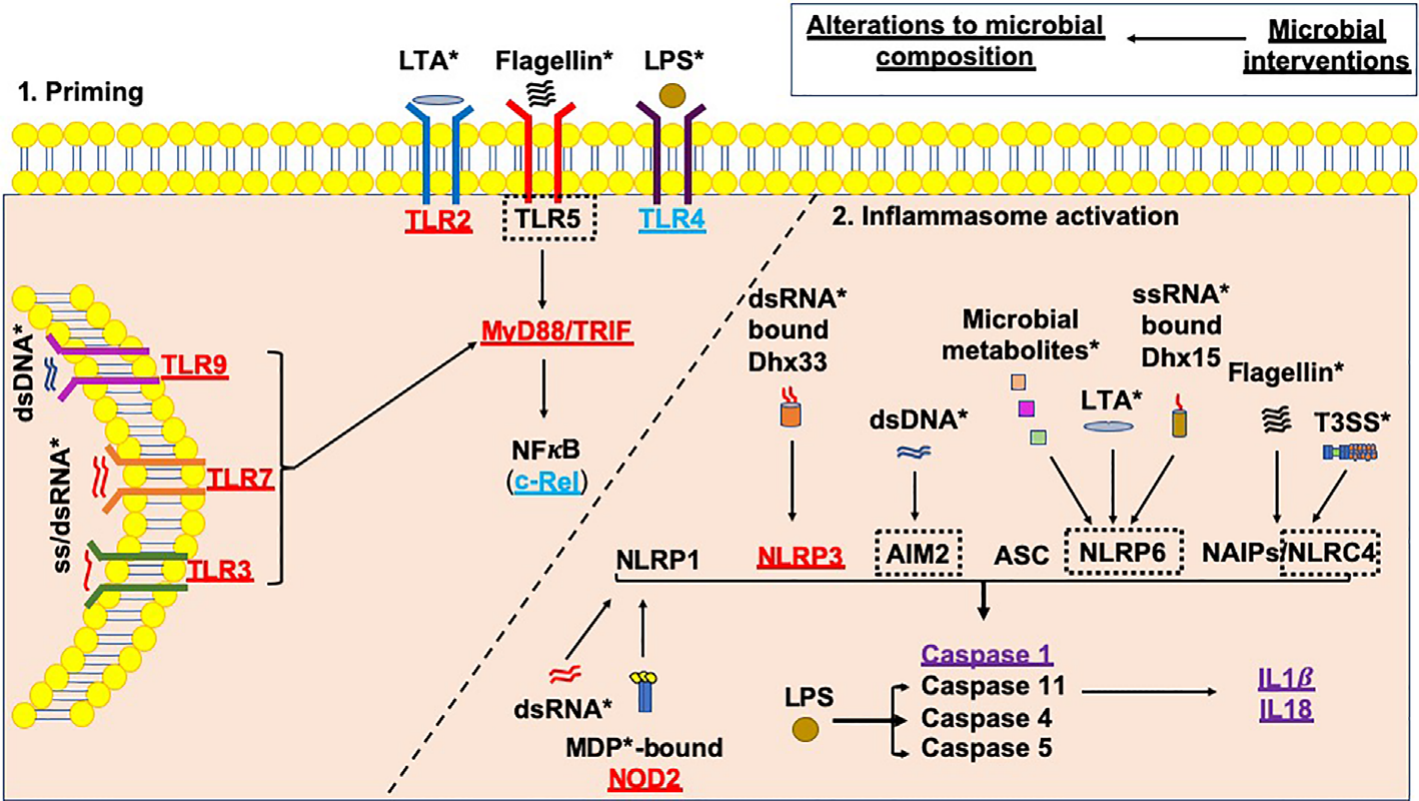
Microbial influences on inflammasome priming and activation in type 1 diabetes. Microbial interventions e.g. fecal microbiota transplants, antibiotic, probiotic and prebiotic usage can all influence the microbial composition, subsequently altering the availability of microbial ligands involved in both the priming, and canonical and non-canonical activation of inflammasomes (as shown by *). Studies of single PRR or inflammasome (*nlrp3*) gene-deficient mice have shown that these proteins would be needed to promote the development of T1D (shown in red); however, Tlr4-deficient and c-Rel-deficient NOD mice (c-Rel is a subunit of the NFкB protein) promote tolerance and limit the development of T1D (shown in blue). In addition, some gene-deficient mice showed no significant effect on mediating susceptibility to T1D (shown in purple). A number of planned studies are currently underway using a number of gene-deficient mice to assess their ability to alter susceptibility to T1D development, as shown by the black dotted boxes. Paradoxically, the gene-deficient mice are also likely to have altered microbial composition, contributing to the protection against/susceptibility to disease. Studies of these gene-deficient mice will need to evaluate the contribution of the gene independently from any alterations to the microbial composition.

Environmental factors, e.g. the microbiota (referring to all microorganisms including bacteria, viruses, fungi, protozoa and archaea), have gained significant traction as modulators of susceptibility to T1D. In turn, it is clear that genes involved in the genetic susceptibility to T1D are important modulators of the bacterial composition in humans and animal models (69, 70). Furthermore, altered gut bacterial composition has been found in individuals diagnosed with T1D (71–75), in Bio-breeding (BB) rats (76), and in Non-obese diabetic (NOD) mice (77, 78), compared to non-diabetic controls. In addition, in individuals who are at genetic risk of developing T1D, changes in gut bacteria are associated with the early development of β-cell autoimmunity (74, 75, 79–81). As mentioned, microbial ligands are one activator of the inflammasomes; changes in the microbial composition and thus the availability of microbial ligands may alter inflammasome activation (**Figure 3**), and this may be one way in which microbes influence pathogenesis of type 1 diabetes.

Viruses have also been implicated in the pathogenesis of T1D. Coxsackie viruses and Rotaviruses have been implicated in the development of T1D due to 1) their association with the development of autoantibodies (82, 83), which are predictive biomarkers for immune progression and T1D development (84); 2) viral proteins e.g. enteroviral capsid protein vp1 can be identified in the islets (85–89); 3) susceptibility to T1D in animal models can be modulated by viral infections (90–98); and 4) an oral Rotavirus vaccine has shown potential to protect individuals at risk of developing T1D from future development of the disease (99). We recently demonstrated that a mouse norovirus infection in NOD mice modulated susceptibility to T1D, mediated through changes in the gut microbiota (100), highlighting the necessity for increased understanding of broader microbial community interactions. Changes in the viral DNA and RNA abundance, alongside any virus-induced bacterial changes, would also potentially alter inflammasome activation.

Both fungal glucans and parasite/helminth antigens can also stimulate inflammasomes and these may modulate susceptibility to T1D in animal models (101–104); however, few studies have been conducted in humans. Individuals with T1D have greater fungal species diversity compared with healthy controls (105). Others demonstrated that individuals with islet autoimmunity, who later progressed to T1D, had a higher abundance of *Sacchromyces* and *Candida*, compared to those who did not progress to T1D over the 8-9 years of follow up (106). There has been much debate about whether parasitic infection modulates autoimmunity in T1D. One study in Norwegian children showed fewer *Enterobius vermicularis* (a pinworm) infections in children at high genetic risk for T1D (107), while another study in Sweden, suggested no association with worms and the development of T1D in children (108). It is possible that parasites may contribute to the reduction in autoimmunity, as parasite-endemic areas have lower incidences of T1D in their populations, compared to non-parasite endemic areas (109). Whilst this may be because parasitic infections promote Th2 immune responses, other factors are likely to be involved including the lower genetic susceptibility to T1D of the populations living in parasite endemic areas. Thus far, although work in animal models has suggested that helminths, and other parasites like schistosomes or their antigenic products (101, 102, 110) could have a beneficial effect on autoimmunity, these have not yet been translated into therapeutics for humans with type 1 diabetes.

Most of the studies mentioned above focus on the microbiota composition and association with the development of either islet autoimmunity or T1D; however, understanding the mechanisms by which the immune system is activated by the microbiota is important. Furthermore, all of these changes in microbial composition may have profound impacts on inflammasome activation (**Figure 3**).

## Inflammasome Priming Is Linked to Type 1 Diabetes Susceptibility

Microbial recognition by PRRs expressed by immune cells are key to regulating crosstalk between immune cells and the microbiota. PRRs such as Toll-like receptors (TLRs), of which there are 10 in humans (TLR1-10) and 12 in mice (TLR1-9, 11-13), selectively bind to their unique microbial ligands, leading to the downstream activation of proinflammatory cytokines (111). These TLRs can be found on different immune and non-immune cells, including the islet β-cells in both humans and mice (112). Studies using TLR-deficient NOD mice have identified that signaling through TLR2, 3, 7 and 9 (97, 113–116) are important for promoting disease, while TLR4 signaling prevents disease development (117). These TLRs signal through one of two key adaptor proteins: Myeloid differentiation primary response 88 (MyD88, which all TLRs utilize except TLR3) or TIR domain-containing adaptor inducing IFN-β (TRIF, which only TLRs 3 and 4 utilize). Deficiencies in either (118, 119), or both (120), of these two key genes results in significant protection of the NOD mice from the development of diabetes, indicating a reliance on downstream-mediated signaling to induce the proinflammatory immune response. Interestingly, only MyD88-deficient mice, but not MyD88 and TRIF double-deficient mice, were protected from immune infiltration in the islets, suggesting that TRIF-mediated signaling, most likely due to TLR4 signaling, was responsible for inducing tolerance (120). TLR4 signaling in human monocyte-derived DCs, stimulated by *E.coli* lipopolysaccharide [LPS; a TLR4 ligand (121)], induced immune tolerance, unlike the effect seen from stimulation with LPS derived from *B.dorei *(122). As Finnish children have a higher abundance of *B.dorei*, and a higher incidence of Type 1 diabetes, compared to their genetically-similar Russian neighbors, it is likely that LPS-induced tolerance is important for modulating susceptibility to T1D in humans (122). TLR activation is also important for priming the inflammasome proteins and thus, changes to the TLR stimulation highlighted above are likely to modulate inflammasome activation as well. It is unclear, at present, whether any of these studies of TLR-deficient mice, or studies of TLR stimulation of cells from individuals with Type 1 diabetes, will differentially influence the activation of the inflammasome and how the functional consequences of this could influence susceptibility to T1D.

In addition to the TLRs, there are also other microbial sensors that can prime the inflammasome complexes, including the cytosolic Nucleotide-binding oligomerization domain (NOD) proteins, NOD1 and NOD2. NOD1 and NOD2 both recognize bacterial peptidoglycan moieties (123, 124) and upon binding, oligomerize and signal through the Receptor-interacting-serine/threonine-protein kinase 2 (RIP2) resulting in the activation of NFκB and production of inflammatory cytokines (125). Using a streptozotocin (STZ)-induced type 1 diabetes model, NOD2 deficiency, but not NOD1 or RIP2 deficiency, protected the mice from disease development (126). These findings were also supported by other studies in NOD mice, demonstrating that NOD2-deficient NOD mice were protected from type 1 diabetes development, and this was dependent on the gut microbiota composition (127), whereas RIP2-deficient NOD mice were not protected (120). Interestingly, both NOD1 and NOD2 appear to have RIP2 independent functions; NOD2 binds CARD9 to mediate downstream signaling independent of RIP2 (128), while NOD1 regulates MAPK signaling independent of RIP2 (129). It is still unclear what the role, if any, NOD1 has in the immunopathogenesis of autoimmune Type 1 diabetes. Importantly, following muramyl dipeptide (ligand) binding, NOD2, complexed with NLRP1, promotes inflammasome activation (33), independent of NOD1 activation (25). Furthermore, in NOD2-deficient mice, induction of intestinal inflammation by dextran sodium sulfate (DSS) resulted in elevated NLRP3 inflammasome formation, suggesting that NOD2 may interact with and/or modulate NLRP3 inflammasome formation (130). Thus, understanding NOD2 activation and its role in modulating inflammasome formation in relation to T1D pathogenesis will need further mechanistic investigation.

It should be noted that in most studies using PRR-deficient NOD mice, the microbiome can be altered by the gene deficiency, which promotes a tolerizing influence and suppression of type 1 diabetes development, as in the case with NOD2-deficient NOD mice (127). Thus, in evaluating studies using these models, it is vital to control for environmental variables such as cage effects (i.e. comparisons between mice in different cages) and legacy effects (i.e. comparisons between mice bred from different breeders), both of which can substantially alter the bacterial composition (131, 132). Failure to consider these variables can promote non-reproducible data and thus future studies need to 1. be transparent in the reporting of these elements in their animal experiments, and 2. Control for these variables.

## Inflammasome Protein Deficiencies Alter Susceptibility to Type 1 Diabetes

To date, only two inflammasome-associated proteins (NLRP3 and AIM2) have been studied for their role in modulating susceptibility to T1D using gene-deficient mice (34, 54, 55). NLRP3-deficient NOD mice were protected from the development of T1D compared to wild-type littermates, as were wild-type NOD mice treated with an NLRP3 inhibitor (parthenolide; 10mg/kg body weight, twice a week for 4 weeks from 10-12 weeks of age) (54). NLRP3-deficient C57BL/6 mice were also protected from diabetes development following STZ treatment, whereas ASC-deficient C57BL/6 mice were not (34). NLRP3 deficiency in NOD mice was found to reduce T cell activation and Th1 differentiation, as well as reducing T cell expression of both the chemokines CCR5 and CXCR3, and *ccl5* and *cxcl10* gene expression from the islet β-cells, resulting in poor T cell chemotaxis into the islets and protection from T1D development (54). Furthermore, diabetic NOD mice exhibited increased *Nlrp3* and *pro-il-1β* gene expression in the pancreatic lymph nodes, compared to pre-diabetic NOD mice, suggesting an increasing role for inflammasome activation (shown to be mediated by circulating mitochondrial DNA) with disease progression (34). In contrast to NLRP3-deficient C57BL/6 mice, AIM2-deficient C57BL/6 mice had accelerated STZ-induced diabetes development, compared to wild-type control mice (55), implying that ASC regulates inflammasome activation. This acceleration in STZ-induced diabetes development in AIM2-deficient mice occurred through enhanced gut permeability and increased bacterial translocation to the pancreatic lymph nodes. These findings were similar to those from the STZ-induced NOD2-deficient mouse study (126), with the inference that NOD2 activation of inflammasomes may be ASC-dependent. In humans, *Aim2* gene expression was increased in the pancreas but not in peripheral blood mononuclear cells (PBMCs) in individuals with T1D compared to healthy controls (55); however, the data from the pancreas was only available in a small group (n=4-8) and thus needs to be confirmed in larger cohorts, ideally separating infiltrating immune cells from the islet β-cells. Another study in humans found that *NLRP1* and *NLRP3* gene expression was reduced in PBMCs and granulocytes in individuals with newly diagnosed T1D (less than 6 months), compared to healthy controls (133). While these studies indicate an important involvement of two of the inflammasome proteins in the development of T1D, further studies are needed to evaluate the other inflammasome-related proteins and how different types of stimulation may influence their function. More studies both in animal models, particularly those developing spontaneous autoimmune diabetes, and in humans, are needed to better understand inflammasome involvement and modulation during diabetes development. Finally, identifying the role of inflammasomes in individual cell types will be pivotal for understanding the key players in inflammasome activation and regulation. Thus, cell-specific gene knock out mice may be valuable tools for such studies.

## Therapeutic Intervention – A Role for Targeting Inflammasomes?

Inflammasome activation induces IL-1β and IL-18 cytokine release following Caspase activation. Both IL-1β and IL-18 cytokines increase with progression to diabetes and destruction of the islet β-cells (134–136). To further investigate whether blocking these pathways could be therapeutically useful, studies targeting the IL-1 pathway were conducted in individuals with recent-onset T1D. Two Phase 2a randomized, multicenter, double-blind, placebo-controlled trials were carried out in which Canakinumab (a human monoclonal anti-IL-1 antibody), or Anakinra (a human IL-1 receptor antagonist), were administered (137). Contrary to expectations, these single immunotherapy interventions failed to prevent the ongoing autoimmunity. This result was concordant with data from NOD mouse models that included IL-1 receptor- (138), Caspase-1- (139, 140), IL-1β- (140) and IL-18- (141)-deficient NOD mice, where no significant changes to diabetes protection were observed with any of these mutations. However, a study combining anti-CD3 treatment with either Anakinra or an anti-IL-1β antibody resulted in reversal of diabetes in recent-onset T1D NOD mice (142), suggesting that combined therapy may also improve clinical efficacy in humans. Given the success of Teplizumab (anti-CD3) in delaying the development of T1D in relatives at risk (143, 144), a combined study evaluating the role of Teplizumab with IL-1 blockade may further enhance clinical efficacy. It is intriguing that NLRP3-deficient NOD mice were protected from T1D, while IL-1 receptor-, Caspase-1/11-, IL-1β- and IL-18-deficient NOD mice were not. There could be multiple reasons for this including: 1. Altered microbiota caused by the gene deficiency, influencing priming/activation of inflammasomes, 2. Promotion of other inflammasome signaling when Nlpr3 is deficient, 3. Effects on other caspases, for example Caspase 8 can also regulate inflammasome activation (145, 146), 4. Effects on other proteases which can process IL-1β (147, 148), and 5. Other unknown protein interactions may be involved. It is clear that further study of multiple pathways of influence is needed to fully comprehend and understand these differences.

Modulation of inflammasomes has had some therapeutic success in autoimmune diseases. A small-molecule inhibitor (MCC950), specifically targeting NLRP3 inflammasome activation (ASC oligomerization) but not AIM2, NLRC4 or NLRP1 inflammasomes, was able to attenuate mouse models of multiple sclerosis (149) and Parkinson’s disease (150). Additional NLRP3 selective inhibitors have been developed, which inhibit ATPase activity (151, 152), or oligomerization of NLRP3 (153), and these inhibitors prevented or ameliorated the development of joint inflammation in arthritis (154), metabolic perturbation in high fat diet-fed mice (151, 153), and autoinflammatory syndromes (151–153). There are also less selective natural inflammasome inhibitors including Genepin, a component of *Gardenis* fruits (155), which can inhibit NLRP3 and NLRC4 inflammasome activation *via* inhibiting autophagy, the eicosanoid 15-deoxy-Δ(12,14)-PGJ2 (15d-PGJ2) and related cyclopentenone prostaglandins (156), which inhibit the NLRP1 and NLRP3 inflammasomes and thence conversion of procaspase 1 to caspase 1. Parthenolide inhibits NLRP1, NLRP3 and NLRC4 inflammasomes (but not AIM2) (157–160), by alkylating the cysteine residues in Caspase 1 and in the ATPase domain of NLRP3 and inhibiting IκB kinase function required for NF-κB activation. As previously mentioned, Parthenolide prevented the development of T1D in 10-12-week old prediabetic NOD mice after 4 weeks of treatment (54). Thus, further investigation of inflammasome inhibitors as a potential therapeutic intervention in T1D is needed. More inflammasome regulators and inhibitors have been studied in different diseases, and which have been reviewed elsewhere (161–163). Future studies should focus on the more selective inflammasome inhibitors, as these will likely have minimal effects on other inflammasome pathways, thereby minimizing detrimental impacts on host defense. Initiating these studies will be vital to fully determine their potential clinical benefits and long-term safety.

Microbes contain multiple ligands that can promote inflammasome activation, thus, therapies targeting the microbiome may also modulate inflammasome responses. Therapies employing microbes or their metabolites have shown some promise in modulating T1D development in animal models (164–168). While supplementation with bacterial-derived short chain fatty acids (SCFAs) protected NOD mice from the development of T1D (164, 168), a human intervention study in which butyrate was administered to longstanding T1D participants was found to have minimal immunological or metabolic effects compared to placebo-treated individuals (169). The human studies were not comparable with the NOD mouse studies however, and further investigation of SCFA administration including dose, duration and timing of treatment should be conducted in those at risk of developing T1D, if the human and mouse investigations are to be compared. In children, early probiotic administration (at the age of 0-27 days) was associated with reduced islet autoimmunity (autoantibodies), compared with children receiving probiotics later than 27 days of age, or those who had never received them (170). A recent study showed that β-cell function could be preserved in newly diagnosed T1D patients, who were recipients of an autologous fecal microbiota transplant, when compared to recipients of an allogeneic (healthy donors) fecal microbiota transplant (171). Together, these studies highlight the potential of harnessing the microbiota as a therapy to modulate ongoing immunity in T1D; however, these studies have not yet evaluated the involvement of the microbial-sensing pathways such as inflammasomes for their ability to modulate the development of diabetes or improved β-cell survival and function.

## Summary

Inflammasomes are important activators of the innate immune response, leading to subsequent adaptive immune responses, particularly in response to microbial ligands. There has been a clear knowledge gap in understanding these inflammasomes in the context of Type 1 diabetes, but more studies are emerging highlighting the importance of the following areas - 1) single nucleotide polymorphisms in inflammasome genes; 2) priming of the inflammasome and 3) the function of the inflammasome proteins in modulating susceptibility to Type 1 diabetes. Together these studies indicate a need to better understand the role of inflammasomes in responding to the microbiota in Type 1 diabetes. At present, to achieve this would require investigators to 1) enlarge the sample sizes for the SNP association studies and investigate the mechanisms behind their association with disease; 2) decipher TLR signaling and inflammasome crosstalk in disease development; 3) investigate how inflammasomes specifically modulate microbial composition and 4) further evaluate inflammasome inhibitors in disease development and how these may be used therapeutically. While this is a new area of investigation, the evidence suggests that studying the inflammasome may provide another possible set of involved pathways that may be amenable to therapeutic targeting to prevent or delay Type 1 diabetes development. Finally, while inflammasomes may have a role in modulating susceptibility to T1D, we should not forget that they are likely to form a part of a multi-mechanistic pathway contributing to the development of T1D. Thus, assessing inflammasome activation in conjunction with other mechanisms of immune activation and regulation may be important to determine a broader picture for clinical interventions.

## Author Contributions

JAP wrote the review. FSW and LW edited the review. All authors contributed to the article and approved the submitted version.

## Funding

This work was funded by a Medical Research Council Career Development Award (MR/T010525/1) to JP, MRC research grant (MR/K021141/1) to SW, and National Institutes of Health (DK 045735, HD 097808), Diabetes Action Research and Education Foundation and Diabetes Research Connection to LW.

## Conflict of Interest

The authors declare that the research was conducted in the absence of any commercial or financial relationships that could be construed as a potential conflict of interest.
